# Palladium(II) complexes of a bridging amine bis­(phenolate) ligand featuring κ^2^ and κ^3^ coordination modes

**DOI:** 10.1107/S2056989019010454

**Published:** 2019-07-26

**Authors:** Brendan J. Graziano, Bradley M. Wile, Matthias Zeller

**Affiliations:** a Donald J. Bettinger Department of Chemistry and Biochemistry, Ohio Northern University, 525 S. Main Street, Ada, Ohio 45810, USA; b Department of Chemistry, Purdue University, 560 Oval Dr., West Lafayette, Indiana 47907, USA

**Keywords:** coordination compound, palladium(II), bidentate & tridentate coordination, amine bis­(phenolate), crystal structure

## Abstract

Bidentate and tridentate coordination of a 2,4-di-*tert*-butyl-substituted bridging amine bis­(phenolate) ligand to a palladium(II) center are observed within the same crystal structure, namely di­chlorido­({6,6′-[(ethane-1,2-diylbis(methyl­aza­nedi­yl)]bis­(methyl­ene)}bis­(2,4-di-*tert*-butyl­phenol))palladium(II) chlorido­(2,4-di-*tert*-butyl-6-{[(2-{[(3,5-di-*tert*-butyl-2-hy­droxy­phen­yl)meth­yl](meth­yl)amino}­eth­yl)(meth­yl)amino]­meth­yl}phenolato)palladium(II) methanol 1.685-solvate 0.315-hydrate, [PdCl_2_(C_34_H_56_N_2_O_2_)][PdCl(C_34_H_55_N_2_O_2_)]·1.685CH_3_OH·0.315H_2_O. Both complexes exhibit a square-planar geometry, with unbound phenol moieties participating in inter­molecular hydrogen bonding with co-crystallized water and methanol. The presence of both κ^2^ and κ^3^ coordination modes arising from the same solution suggest a dynamic process in which phenol donors may coordinate or dissociate from the metal center, and offers insight into catalyst speciation throughout Pd-mediated processes. The unit cell contains di­chlorido­({6,6′-[(ethane-1,2-diylbis(methyl­aza­nedi­yl)]bis­(methyl­ene)}bis­(2,4-di-*tert*-butyl­phenol))palladium(II), {(*L*_2_)PdCl_2_}, and chlorido­(2,4-di-*tert*-butyl-6-{[(2-{[(3,5-di-*tert*-butyl-2-hy­droxy­phen­yl)meth­yl](methyl)amino}eth­yl)(meth­yl)amino]­meth­yl}phenolato)palladium(II), {(*L*_2_ *X*)PdCl}, mol­ecules as well as fractional water and methanol solvent mol­ecules.

## Abstract 2

The crystal structure of a palladium(II) coordination compound features the same ligand bound to the metal center in both a κ^2^ and κ^3^ fashion.

## Chemical context   

The activity of early transition-metal and rare-earth complexes of amine bis­(phenolate) ligands for olefin (Tshuva *et al.*, 2000 ▸) and cyclic ester polymerization (Carpentier, 2015 ▸) has been well documented. Several studies (Tshuva *et al.*, 2001 ▸; Qian *et al.*, 2011 ▸) demonstrated that the coordination mode and donor identity play a significant role in the activity of complexes derived from amine bis­(phenolate) and related ligands. Amine bis­(phenolate) complexes of iron have been employed as catalysts for cross-coupling (Chowdhury *et al.*, 2008 ▸), polymerization (Allan *et al.*, 2014 ▸) and CO_2_ conversion (Andrea *et al.*, 2018 ▸) and as functional models for various non-heme metalloenzymes (Karimpour *et al.*, 2013 ▸; Strautmann *et al.*, 2011 ▸). While a relatively limited number of late transition-metal amine bis­(phenolate) complexes have been employed as catalysts, nearly all have been observed to bind through both amine and both phenolate donor atoms to form κ^4^ complexes. Related complexes featuring κ^2^ or κ^3^ coordination modes may offer unique insight into catalyst identity for species that may not be directly observed.

Several related complexes feature ligands similar to these amine bis­(phenolate) species bound in a κ^3^ fashion. Notably, Zn phen­oxy di­amine complexes are highly active catalysts for the polymerization of lactide. (Williams *et al.*, 2003 ▸; Labourdette *et al.*, 2009 ▸) Related modification to the amine bis­(phenolate) framework generated ‘claw-type’ κ^3^ Zn (Song *et al.*, 2012 ▸; Wang *et al.*, 2010 ▸) and Ti (Zhao *et al.*, 2014 ▸) complexes that serve as competent polymerization catalysts. To our knowledge, only one report describes Pd complexes with amine bis­(phenolate) ligands bound in a κ^2^ or κ^3^ coord­ination mode, in which both amine donors remain bound, and one phenolate donor may bind to the Pd center (Graziano *et al.*, 2019 ▸). These species exhibit coordination behavior that varies with the steric parameters of the phenolate *ortho* and *para* substituents, with larger cumyl substituents favoring the formation of κ^2^ complexes. In this work, we describe diffraction data for a related Pd complex featuring the ligand {6,6′-[(ethane-1,2-diylbis(methyl­aza­nedi­yl)]bis­(methyl­ene)}bis­(2,4-di-*tert*-butyl­phenol) bound in both κ^2^ or κ^3^ coordination modes within a single unit cell. The presence of palladium(II) complexes displaying both κ^2^ and κ^3^ coordination modes arising from the same solution suggests a dynamic process in which phenol donors may coordinate or de-coordinate based on the electronic demands at the metal center.
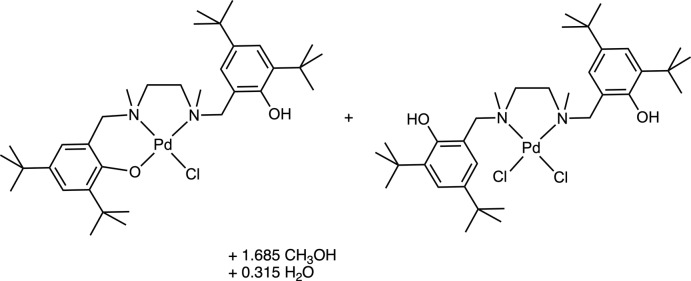


## Structural commentary   

**Figure 1:**
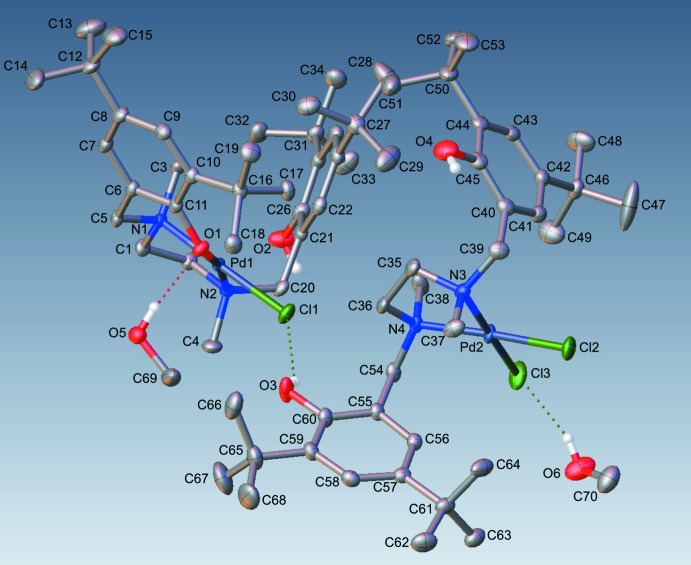
The asymmetric unit of the title compound with only the major components of disorder shown. Displacement ellipsoids are drawn at the 50% probability level. H atoms bonded to C atoms are omitted for clarity and hydrogen bonds are shown as dotted lines.

The asymmetric unit of the structure (Fig. 1 ▸) consists of two distinct palladium(II) complexes of the amine bis­(phenolate) {6,6′-[(ethane-1,2-diylbis(methyl­aza­nedi­yl)]bis­(methyl­ene)}bis­(2,4-di-*tert*-butyl­phenol) and fractional qu­anti­ties of methanol and water crystallization solvents. Both metal centers adopt similar distorted square-planar geometric arrangements, in which both nitro­gen atoms of the ligand are bound to the Pd center to form a five-membered ring, and either one or two chlorine atoms are present to complete the coordination sphere depending on the coordination mode of the ligand. In both complexes, the N—Pd—N bond angle is similar to those observed for related amine bis­(phenolate) Pd complexes (Graziano *et al.*, 2019 ▸), as described in Table 1 ▸.

Deprotonation and coordination of O1, presumably in the presence of water during crystallization, gives rise to the [(κ^3^-*N*,*N*,*O*)PdCl] complex. This complex is only slightly distorted from ideal square-planar geometry (τ_4_ parameter = 0.0823; Yang *et al.*, 2007 ▸), and Pd1 lies 0.073 Å above the plane defined by O1/N1/N2/Cl1. The phenol ring containing O2 is disordered by rotation about the C20—C21 bond, such that in the minor component a close O2*B*—H2*C*⋯Cl1 inter­action of ∼2.065 Å is observed (see *Refinement* section for details of the disorder). An additional close contact of ∼2.460 Å is observed between Cl1 and the O3—H bond of an unbound phenol from the neighboring [(κ^2^-*N*,*N*)PdCl_2_] complex.

The [(κ^2^-*N*,*N*)PdCl_2_] complex also exhibits only minor distortions from an ideal square-planar geometry (τ_4_ parameter = 0.0638; Yang *et al.*, 2007 ▸), and Pd2 lies within (±0.001 Å) the plane defined by N3/N4/Cl2/Cl3. Both phenol rings in this complex are disordered by rotation about the C54—C55 bond and the C39—C40 bond, giving rise to a close O3*B*—H3*D*⋯Cl3 inter­action of ∼2.12 Å and an O4*B*—H4*D*⋯Cl2 inter­action of ∼2.05 Å, respectively.

Solution NMR data (see *Synthesis and crystallization* section) suggest that the conformations observed in the solid state are retained on the NMR timescale. Signals attributed to both the κ^2^ and κ^3^ complexes are observed, including signals attributed to the protonated phenol moieties. Signals attributed to the ligand methyl­ene groups are rendered diastereotopic upon coordination of the distal donor atoms to the Pd center, while methyl­ene units for unbound donors remain magnetically equivalent.

## Supra­molecular features   

**Figure 2:**
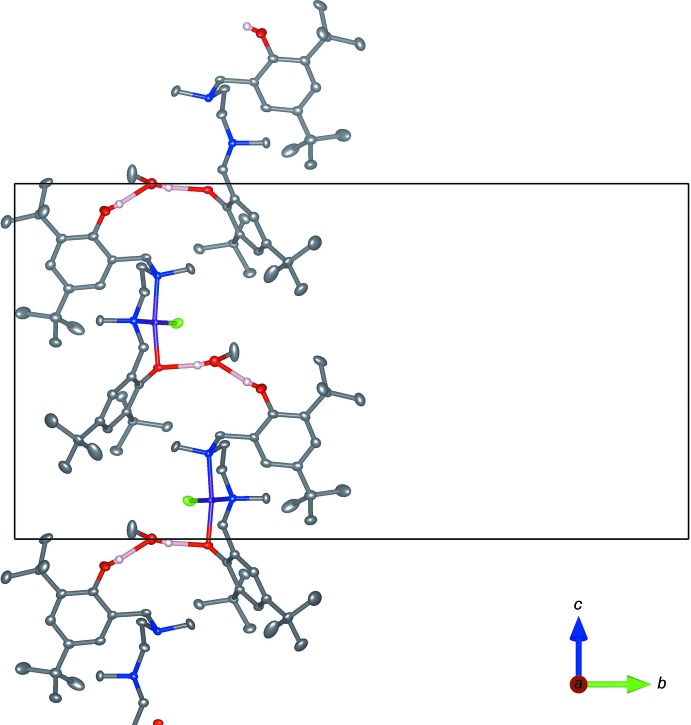
Part of the crystal structure viewed along the *a* axis, showing hydrogen bonding (in pink) between neighboring κ^3^ amine bis­(phenolate) Pd^II^ complexes.

Hydrogen bonding (Table 2 ▸) is observed between phenol O2—H2, co-crystallized methanol solvent O5—H5, and O1 of a neighboring complex, forming a two-dimensional network in the *bc* plane between [(κ^3^-*N*,*N*,*O*)PdCl] subunits. Details of this inter­action are illustrated in Fig. 2 ▸, which depicts the inter­action between neighboring κ^3^ species, viewed along the *a* axis. Additional O—H⋯Cl inter­actions are observed between O3—H3 and Cl1, and O6—H6 and Cl3, though neither of these inter­actions forms an extended network. The inter­action between O3—H3 and Cl1 is of inter­est as it is the only observed close contact between the κ^2^ and κ^3^ complexes within the asymmetric unit. This feature is absent in the minor component, in which the [(κ^2^-*N*,*N*)PdCl_2_] phenol hy­droxy moiety O3*B*—H3*D* exhibits an intra­molecular close contact with Cl3. Within the minor component, a related intra­molecular close contact is observed between the remaining [(κ^2^-*N*,*N*)PdCl_2_] phenol hy­droxy group O4*B—*H4*D* and Cl2.

## Synthesis and crystallization   

**Figure 3:**
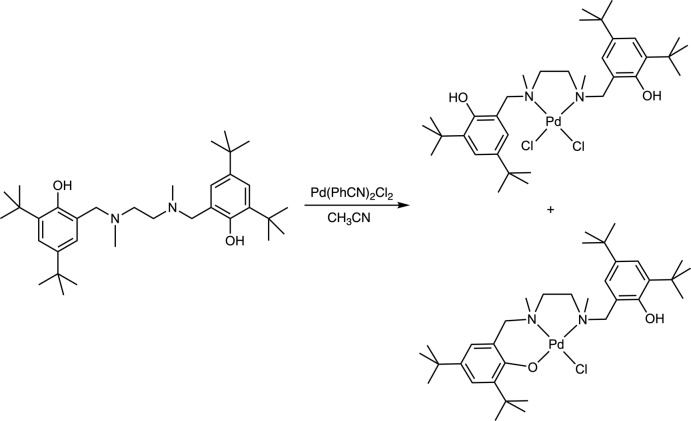
Preparation of κ^2^ and κ^3^ amine bis­(phenolate) Pd^II^ complexes.

Both species within this unit cell are generated upon combining equimolar qu­anti­ties (0.254 mmol) of the {6,6′-[(ethane-1,2-diylbis(methyl­aza­nedi­yl)]bis­(methyl­ene)}bis­(2,4-di-*tert*-butyl­phenol) ligand and bis­(benzo­nitrile)­dichloro­palladium(II) in 5 mL of aceto­nitrile, using the method reported previously by Wile and co-workers (Graziano *et al.*, 2019 ▸) as shown in Fig. 3 ▸. The titular compound was obtained as an orange solid (116 mg, 0.169 mmol, 67%). Single crystals suitable for X-ray diffraction studies were grown from a concentrated solution of the metal complex in methanol, layered with distilled water (∼10:1 *v*/*v*).

^1^H and ^13^C NMR spectra reveal signals attributed to both κ^2^ and κ^3^ Pd complexes in CDCl_3_ solution. The equilibrium, and the position of several signals shifts slightly when CD_3_OD is employed as the solvent for NMR characterization. The cleanest spectral data were obtained in CDCl_3_, and are reported below. Upon coordination, several methyl­ene H’s were rendered diastereotopic. Spectroscopic assignments were confirmed through the use of 2D NMR (COSY, HSQC, HMBC) and polarization transfer (DEPT-135) experiments. ^1^H (CDCl_3_, 400.132 MHz) δ = 8.05 (*s*, 1H, OH), 7.51 (*s*, 1H, aryl C-H), 7.42 (*m*, 1H, aryl C-H), 7.36 (*m*, 2H, aryl C-H), 7.17–7.08 (*m*, 2H, aryl C-H), 6.83 (*s*, 1H, OH), 6.55 (*m*, 1H, aryl C-H), 4.64–4.50 (*m*, 2H, CH_2_), 3.79–3.65 (*m*, 1H, CH_2_), 3.59–3.48 (*m*, 1H, CH_2_), 3.35–3.20 (*m*, 3H), 3.00 (*d*, *J* = 13.6 Hz, 1H, CH_2_), 2.92–2.80 (*m*, 2H, CH_2_), 2.73–2.64 (*m*, 1H, CH_2_), 2.47 (*s*, 1H), 2.37–2.29 (*m*, 1H, CH_2_), 2.19–2.10 (*m*, 1H, CH_2_), 1.62 (*s*, 3H, CH_3_), 1.50 (*s*, 3H, CH_3_), 1.47–1.38 (*m*, 15H, CH_3_), 1.32 (*s*, 6H, CH_3_), 1.30 (*s*, 3H, CH_3_), 1.27 (*s*, 6H, CH_3_), 1.19 (*s*, 6H, CH_3_); ^13^C{^1^H} (CDCl_3_, 100.613 MHz) δ = 158.1 (4°), 153.3 (4°), 141.6 (4°), 139.6 (4°), 136.3 (4°), 129.0 (aryl C-H), 128.7 (4°), 125.5 (4°), 124.9 (4°), 124.0 (aryl C-H), 121.0 (4°), 117.6 (4°), 65.5 (CH_2_), 62.6 (CH_2_), 62.5 (CH_2_), 61.1 (CH_2_), 55.8 (CH_2_), 51.5, 41.7 (CH_3_), 35.4 (^*t*^Bu 4°), 35.3 (^*t*^Bu 4°), 35.0 (^*t*^Bu 4°), 34.3 (^*t*^Bu 4°), 34.1 (^*t*^Bu 4°), 33.8 (^*t*^Bu 4°), 31.6 (CH_3_), 30.2 (CH_3_), 30.1 (CH_3_), 29.9 (CH_3_), 29.6 (CH_3_). m.p. 435 K (decomp.)

## Refinement   

Crystal data, data collection and structure refinement details are summarized in Table 3 ▸.

Water H atoms were restrained to have O—H bond lengths of 0.84 (2) Å, and 1.36 (2) Å H⋯H distances (DFIX, esd = 0.02 Å). All H atoms attached to carbon atoms as well as phenol and methanol hydroxyl hydrogens were positioned geometrically and constrained to ride on their parent atoms. C—H bond distances were constrained to 0.95 Å for aromatic C—H moieties, and to 0.99 and 0.98 Å for aliphatic CH_2_ and CH_3_ moieties, respectively. Phenol and methanol O—H distances were constrained to 0.84 Å. Methyl CH_3_ and hydroxyl H atoms were allowed to rotate but not to tip to best fit the experimental electron density. *U*_iso_(H) values were set to a multiple of *U*_eq_(C/O) with 1.5 for CH_3_, OH and water, and 1.2 for C—H, CH_2_, units, respectively.

Three of the four phenol hydroxyl groups are positionally disordered by rotation of the aromatic ring. For two of the three minor moieties, the O—C distance and the 1,3 O to C distances of the minor and major moieties were restrained to be similar (SADI command of *SHELX*, esd = 0.02 Å). Minor O atom O2*B* was constrained to have the same ADP as the C atom to which it is bonded. Two phenol H-atom positions were positionally restrained based on hydrogen-bonding considerations and to avoid close contacts to C-bound H atoms. Subject to these conditions, the occupancy rates of the major moieties refined to 0.917 (3), 0.857 (4) and 0.899 (4).

A *tert-*butyl group was refined as rotationally disordered. The two moieties were restrained to have similar geometries, the central C atoms to share one ADP, and the *U* ^ij^ components of ADPs were restrained to be similar (SIMU command of *SHELX*, esd = 0.01 Å^2^). Subject to these conditions the occupancy ratio refined to 0.716 (8):0.284 (8).

One solvate methanol mol­ecule was refined as disordered over two orientations, and another to be disordered with a water mol­ecule. The O—C distances of the major and minor methanol mol­ecules were restrained to be similar (SADI, esd = 0.02 Å), and the *U* ^ij^ components of ADPs were restrained to be similar for the three methanol and the one water moiety (SIMU, esd = 0.01 Å^2^). Subject to these conditions the occupancy ratio refined to 0.685 (8):0.315 (8) for the methanol-to-water ratio, and 0.843 (4):0.157 (4) for the methanol-to-methanol ratio.

## Supplementary Material

Crystal structure: contains datablock(s) I, global. DOI: 10.1107/S2056989019010454/lh5914sup1.cif

Structure factors: contains datablock(s) I. DOI: 10.1107/S2056989019010454/lh5914Isup2.hkl

CCDC reference: 1940199

Additional supporting information:  crystallographic information; 3D view; checkCIF report

## Figures and Tables

**Table 1:** N—Pd—N bond angles for amine bis­(phenolate) Pd^II^ complexes

Complex	Reference	N—Pd—N
[(κ^2^-*N*,*N*)PdCl_2_]	This work	85.44
[(κ^3^-*N*,*N*,*O*)PdCl]	This work	85.84
[(κ^2^-*N*,*N*)PdCl_2_]	Graziano *et al.* (2019 ▸)	82.8
[(κ^2^-*N*,*N*)PdCl_2_]	Graziano *et al.* (2019 ▸)	85.58
[(κ^3^-*N*,*N*,*O*)PdCl]	Graziano *et al.* (2019 ▸)	82.82
[(κ^3^-*N*,*N*,*O*)PdCl]	Graziano *et al.* (2019 ▸)	86.86
[(κ^3^-*N*,*N*,*O*)PdCl]	Graziano *et al.* (2019 ▸)	82.9
[(κ^3^-*N*,*N*,*O*)PdCl]	Graziano *et al.* (2019 ▸)	83.0
[(κ^2^-*N*,*N*,)PdCl_2_]	Ding *et al.* (2011 ▸)	84.07

**Table 2:** Hydrogen-bond geometry (Å, °)

*D*—H⋯*A*	*D*—H	H⋯*A*	*D*⋯*A*	*D*—H⋯*A*
O5—H5⋯O1	0.84	1.95	2.787 (3)	171
C69—H69*A*⋯Cl1	0.98	2.90	3.642 (4)	134
C69—H69*C*⋯O3	0.98	2.57	3.267 (4)	128
O5*B*—H5*C*⋯O1	0.84 (2)	1.95 (3)	2.772 (6)	168 (12)
O6—H6⋯Cl2	0.84	2.90	3.592 (2)	141
O6—H6⋯Cl3	0.84	2.41	3.129 (2)	144
O6*B*—H6*B*⋯Cl2	0.84	2.45	3.242 (13)	157
O2—H2⋯O5^i^	0.84	1.88	2.715 (3)	170
O2*B*—H2*C*⋯Cl1	0.84	2.06	2.858 (13)	157
O3—H3⋯Cl1	0.84	2.46	3.1764 (17)	144
O3*B*—H3*D*⋯Cl3	0.84	2.12	2.886 (10)	152
O4—H4⋯O6^ii^	0.84	1.95	2.750 (3)	160
O4*B*—H4*D*⋯Cl2	0.84	2.05	2.726 (12)	137

Symmetry codes: (i) 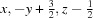; (ii) 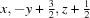.

**Table 3:** Experimental details

Crystal data	
Chemical formula	[PdCl_2_(C_34_H_56_N_2_O_2_)][PdCl(C_34_H_55_N_2_O_2_)]·1.685CH_4_O·0.315H_2_O
*M*_r_	1427.41
Crystal system, space group	Monoclinic, *P*2_1_/*c*
Temperature (K)	100
*a*, *b*, *c* (Å)	15.8843 (5), 29.780 (1), 16.6629 (6)
β (°)	109.5536 (12)
*V* (Å^3^)	7427.6 (4)
*Z*	4
Radiation type	Mo *K*α
μ (mm^−1^)	0.64
Crystal size (mm)	0.42 × 0.27 × 0.09

Data collection	
Diffractometer	Bruker AXS D8 Quest CMOS
Absorption correction	Multi-scan (*SADABS*; Krause *et al.*, 2015 ▸)
*T*_min_, *T*_max_	0.656, 0.747
No. of measured, independent and observed [*I* > 2σ(*I*)] reflections	87447, 22272, 18396
*R*_int_	0.041
(sin θ/λ)_max_ (Å^−1^)	0.714

Refinement	
*R*[*F* ^2^ > 2σ(*F* ^2^)], *wR*(*F* ^2^), *S*	0.033, 0.076, 1.03
No. of reflections	22272
No. of parameters	900
No. of restraints	186
H-atom treatment	H atoms treated by a mixture of independent and constrained refinement
Δρ_max_, Δρ_min_ (e Å^−3^)	2.12, −0.92

Computer programs: *APEX3* and *SAINT* (Bruker, 2016 ▸), *SHELXS97* (Sheldrick, 2008 ▸), *SHELXL2016/6* (Sheldrick, 2015 ▸), *SHELXLE* (Hübschle *et al.*, 2011 ▸), *OLEX2* (Dolomanov, 2009 ▸), *VESTA* (Momma & Izumi, 2011 ▸) and *publCIF* (Westrip, 2010 ▸).

## supplementary crystallographic information

### Crystal data

**Table:**

[PdCl_2_(C_34_H_56_N_2_O_2_)][PdCl(C_34_H_55_N_2_O_2_)]·1.685CH_4_O·0.315H_2_O	*F*(000) = 3022
*M**_r_* = 1427.41	*D*_x_ = 1.276 Mg m^−^^3^
Monoclinic, *P*2_1_/*c*	Mo *K*α radiation, λ = 0.71073 Å
*a* = 15.8843 (5) Å	Cell parameters from 9425 reflections
*b* = 29.780 (1) Å	θ = 2.9–36.3°
*c* = 16.6629 (6) Å	µ = 0.64 mm^−^^1^
β = 109.5536 (12)°	*T* = 100 K
*V* = 7427.6 (4) Å^3^	Plate, orange
*Z* = 4	0.42 × 0.27 × 0.09 mm

### Data collection

**Table:**

Bruker AXS D8 Quest CMOS diffractometer	22272 independent reflections
Radiation source: sealed tube X-ray source	18396 reflections with *I* > 2σ(*I*)
Triumph curved graphite crystal monochromator	*R*_int_ = 0.041
ω and phi scans	θ_max_ = 30.5°, θ_min_ = 2.9°
Absorption correction: multi-scan (SADABS; Krause *et al.*, 2015)	*h* = −22→22
*T*_min_ = 0.656, *T*_max_ = 0.747	*k* = −42→35
87447 measured reflections	*l* = −18→23

### Refinement

**Table:**

Refinement on *F*^2^	Primary atom site location: structure-invariant direct methods
Least-squares matrix: full	Secondary atom site location: difference Fourier map
*R*[*F*^2^ > 2σ(*F*^2^)] = 0.033	Hydrogen site location: mixed
*wR*(*F*^2^) = 0.076	H atoms treated by a mixture of independent and constrained refinement
*S* = 1.03	*w* = 1/[σ^2^(*F*_o_^2^) + (0.0251*P*)^2^ + 7.3036*P*] where *P* = (*F*_o_^2^ + 2*F*_c_^2^)/3
22272 reflections	(Δ/σ)_max_ = 0.006
900 parameters	Δρ_max_ = 2.12 e Å^−^^3^
186 restraints	Δρ_min_ = −0.92 e Å^−^^3^

### Special details

**Table:**

Geometry. All esds (except the esd in the dihedral angle between two l.s. planes) are estimated using the full covariance matrix. The cell esds are taken into account individually in the estimation of esds in distances, angles and torsion angles; correlations between esds in cell parameters are only used when they are defined by crystal symmetry. An approximate (isotropic) treatment of cell esds is used for estimating esds involving l.s. planes.
Refinement. Three of the four phenol hydroxyl groups are positionally disordered by rotation of the aromatic ring. For two of the three minor moieties the O-C distance and the 1,3 O to C distances of the minor and major moieties were restrained to be similar. Minor O atom O2B was constrained to have the same ADP as the C atom it is bonded to. Two phenol H atom positions were positionally restrained based on H-bonding considerations and to avoid close contacts to C bound H atoms. Subject to these conditions the occupancy rates of the major moieties refined to 0.917 (3), 0.857 (4) and 0.899 (4). A tert butyl group was refined as rotationally disordered. The two moieties were restrained to have similar geometries, the central C atoms to share one ADP, and Uij components of ADPs were restrained to be similar. Subject to these conditions the occupancy ratio refined to 0.716 (8) to 0.284 (8). One solvate methanol molecule was refined as disordered over two orientations, and another to be disordered with a water molecule. The O-C distance of the major and minor methanol molecules were restrained to be similar, and Uij components of ADPs were restrained to be similar for the three methanol and the one water moiety. Water H atoms were restrained to have O-H bond lengths of 0.84 (2) Angstroms, and 1.36 (2) Angstrom H···H distances. Subject to these conditions the occupancy ratio refined to 0.685 (8) to 0.315 (8) for the methanol to water ratio, and 0.843 (4) to 0.157 (4) for the methanol to methanol ratio.

### Fractional atomic coordinates and isotropic or equivalent isotropic displacement parameters (Å^2^)

**Table:**

	*x*	*y*	*z*	*U*_iso_*/*U*_eq_	Occ. (<1)
C1	0.17953 (11)	0.80735 (7)	0.30459 (11)	0.0194 (3)	
H1A	0.124296	0.825478	0.291263	0.023*	
H1B	0.164217	0.775609	0.310792	0.023*	
C2	0.21826 (11)	0.81161 (6)	0.23312 (11)	0.0186 (3)	
H2A	0.177086	0.797913	0.180441	0.022*	
H2B	0.225933	0.843696	0.221660	0.022*	
C3	0.25578 (12)	0.87326 (6)	0.38525 (12)	0.0189 (3)	
H3A	0.274902	0.882313	0.337477	0.028*	
H3B	0.300237	0.883118	0.438856	0.028*	
H3C	0.197988	0.887119	0.379094	0.028*	
C4	0.29176 (14)	0.73906 (6)	0.23957 (13)	0.0252 (4)	
H4A	0.259934	0.735029	0.178526	0.038*	
H4B	0.255957	0.726570	0.272035	0.038*	
H4C	0.349382	0.723493	0.255466	0.038*	
C5	0.22069 (11)	0.80871 (6)	0.46069 (11)	0.0175 (3)	
H5A	0.159724	0.819662	0.453394	0.021*	
H5B	0.219857	0.775496	0.462801	0.021*	
C6	0.28488 (11)	0.82650 (6)	0.54299 (11)	0.0172 (3)	
C7	0.25435 (12)	0.85501 (6)	0.59361 (12)	0.0203 (3)	
H7	0.192537	0.862079	0.576693	0.024*	
C8	0.31289 (13)	0.87344 (6)	0.66884 (12)	0.0214 (4)	
C9	0.40305 (12)	0.86206 (6)	0.69068 (11)	0.0195 (3)	
H9	0.443524	0.874213	0.741761	0.023*	
C10	0.43751 (11)	0.83389 (6)	0.64207 (11)	0.0176 (3)	
C11	0.37664 (11)	0.81525 (6)	0.56647 (11)	0.0165 (3)	
C12	0.27754 (14)	0.90546 (7)	0.72233 (13)	0.0278 (4)	
C13	0.2301 (2)	0.94537 (9)	0.66700 (18)	0.0493 (7)	
H13A	0.271220	0.960154	0.642776	0.074*	
H13B	0.211116	0.966851	0.702121	0.074*	
H13C	0.177649	0.934537	0.620839	0.074*	
C14	0.2107 (2)	0.88091 (10)	0.75446 (19)	0.0514 (7)	
H14A	0.162850	0.868296	0.705981	0.077*	
H14B	0.185163	0.902034	0.785119	0.077*	
H14C	0.241205	0.856620	0.792902	0.077*	
C15	0.35247 (18)	0.92506 (9)	0.79799 (15)	0.0401 (6)	
H15A	0.394948	0.941150	0.777254	0.060*	
H15B	0.383447	0.900697	0.835955	0.060*	
H15C	0.326986	0.945892	0.829225	0.060*	
C16	0.53860 (11)	0.82428 (6)	0.66905 (11)	0.0190 (3)	
C17	0.57415 (12)	0.84092 (7)	0.59878 (12)	0.0238 (4)	
H17A	0.544245	0.824557	0.545847	0.036*	
H17B	0.638691	0.835647	0.616394	0.036*	
H17C	0.562100	0.873116	0.589428	0.036*	
C18	0.55738 (13)	0.77371 (7)	0.68368 (13)	0.0239 (4)	
H18A	0.525242	0.757245	0.631485	0.036*	
H18B	0.537201	0.763440	0.730096	0.036*	
H18C	0.621664	0.768247	0.698789	0.036*	
C19	0.59150 (13)	0.84892 (8)	0.75145 (13)	0.0289 (4)	
H19A	0.655086	0.841619	0.766644	0.043*	
H19B	0.569987	0.839469	0.797507	0.043*	
H19C	0.583179	0.881385	0.742794	0.043*	
C20	0.36366 (12)	0.80514 (6)	0.20834 (11)	0.0194 (3)	
H20A	0.422187	0.789679	0.228461	0.023*	
H20B	0.334165	0.797097	0.147732	0.023*	
C21	0.37937 (12)	0.85519 (6)	0.21469 (11)	0.0179 (3)	
C22	0.44292 (12)	0.87334 (7)	0.28698 (11)	0.0201 (3)	
H22	0.476336	0.853829	0.331313	0.024*	0.917 (3)
C23	0.45843 (12)	0.91936 (7)	0.29554 (12)	0.0216 (4)	
C24	0.40753 (12)	0.94681 (7)	0.22901 (12)	0.0224 (4)	
H24	0.416930	0.978327	0.234504	0.027*	
C25	0.34364 (12)	0.93061 (7)	0.15490 (12)	0.0209 (4)	
C26	0.33134 (12)	0.88384 (7)	0.14817 (11)	0.0213 (4)	
H26	0.289926	0.871434	0.097885	0.026*	0.083 (3)
C27	0.52639 (14)	0.93896 (7)	0.37683 (13)	0.0274 (4)	
C28	0.5518 (2)	0.98740 (10)	0.36385 (17)	0.0528 (8)	
H28A	0.577123	0.988309	0.317726	0.079*	
H28B	0.596057	0.998444	0.416539	0.079*	
H28C	0.498403	1.006414	0.348971	0.079*	
C29	0.61076 (15)	0.90945 (10)	0.40685 (17)	0.0475 (7)	
H29A	0.594515	0.879079	0.418959	0.071*	
H29B	0.653492	0.922361	0.458601	0.071*	
H29C	0.637970	0.908136	0.362089	0.071*	
C30	0.48404 (16)	0.93941 (8)	0.44706 (13)	0.0320 (5)	
H30A	0.431519	0.959059	0.430062	0.048*	
H30B	0.527586	0.950598	0.500060	0.048*	
H30C	0.465948	0.908872	0.455959	0.048*	
C31	0.28669 (13)	0.96291 (7)	0.08547 (13)	0.0235 (4)	
C32	0.18826 (14)	0.95698 (8)	0.07806 (15)	0.0310 (4)	
H32A	0.151257	0.978120	0.035828	0.047*	
H32B	0.181486	0.962839	0.133454	0.047*	
H32C	0.169280	0.926184	0.060189	0.047*	
C33	0.29709 (16)	0.95343 (8)	−0.00108 (13)	0.0342 (5)	
H33A	0.257826	0.973494	−0.044085	0.051*	
H33B	0.280830	0.922156	−0.017292	0.051*	
H33C	0.359260	0.958571	0.002776	0.051*	
C34	0.31216 (15)	1.01223 (7)	0.10743 (15)	0.0335 (5)	
H34A	0.274848	1.031531	0.061685	0.050*	
H34B	0.375168	1.016687	0.113673	0.050*	
H34C	0.302732	1.019955	0.160962	0.050*	
C35	0.82670 (13)	0.78738 (7)	0.62727 (12)	0.0227 (4)	
H35A	0.824884	0.784692	0.685884	0.027*	
H35B	0.814599	0.819066	0.609060	0.027*	
C36	0.75680 (12)	0.75753 (6)	0.56829 (12)	0.0214 (4)	
H36A	0.763320	0.726637	0.591504	0.026*	
H36B	0.696603	0.768609	0.563713	0.026*	
C37	0.94963 (15)	0.73294 (7)	0.67850 (12)	0.0260 (4)	
H37A	0.906169	0.708645	0.657586	0.039*	
H37B	0.956574	0.739453	0.738028	0.039*	
H37C	1.007331	0.723757	0.674484	0.039*	
C38	0.72900 (13)	0.79913 (7)	0.43422 (14)	0.0276 (4)	
H38A	0.733626	0.797676	0.377086	0.041*	
H38B	0.762670	0.825093	0.464726	0.041*	
H38C	0.666107	0.802153	0.429580	0.041*	
C39	0.98438 (13)	0.81102 (7)	0.66335 (12)	0.0242 (4)	
H39A	0.994263	0.813458	0.725091	0.029*	
H39B	1.042106	0.803016	0.656532	0.029*	
C40	0.95395 (12)	0.85599 (7)	0.62173 (12)	0.0223 (4)	
C41	0.96205 (12)	0.86495 (7)	0.54242 (12)	0.0216 (4)	
H41	0.992642	0.844110	0.518897	0.026*	0.899 (4)
C42	0.92636 (12)	0.90370 (6)	0.49693 (12)	0.0206 (3)	
C43	0.88280 (12)	0.93363 (6)	0.53447 (12)	0.0220 (4)	
H43	0.856925	0.959867	0.503581	0.026*	
C44	0.87513 (12)	0.92721 (6)	0.61466 (12)	0.0230 (4)	
C45	0.91311 (13)	0.88776 (7)	0.65884 (12)	0.0247 (4)	
H45	0.911017	0.882651	0.714420	0.030*	0.101 (4)
C46	0.9314 (4)	0.9110 (2)	0.4058 (6)	0.0242 (7)	0.716 (8)
C47	1.0265 (4)	0.9038 (3)	0.4074 (6)	0.083 (3)	0.716 (8)
H47A	1.029465	0.908772	0.350239	0.125*	0.716 (8)
H47B	1.045109	0.872976	0.425470	0.125*	0.716 (8)
H47C	1.066331	0.924959	0.447385	0.125*	0.716 (8)
C48	0.9003 (4)	0.95804 (12)	0.3720 (3)	0.0453 (12)	0.716 (8)
H48A	0.905189	0.961543	0.315213	0.068*	0.716 (8)
H48B	0.937886	0.980505	0.410573	0.068*	0.716 (8)
H48C	0.837993	0.962226	0.368466	0.068*	0.716 (8)
C49	0.8698 (3)	0.87713 (15)	0.3444 (2)	0.0431 (11)	0.716 (8)
H49A	0.808235	0.881608	0.343165	0.065*	0.716 (8)
H49B	0.889015	0.846534	0.363541	0.065*	0.716 (8)
H49C	0.872653	0.881615	0.287067	0.065*	0.716 (8)
C46B	0.9326 (11)	0.9135 (5)	0.4130 (16)	0.0242 (7)	0.284 (8)
C47B	1.0007 (11)	0.8840 (6)	0.3924 (10)	0.060 (5)	0.284 (8)
H47D	1.005037	0.892881	0.337305	0.090*	0.284 (8)
H47E	0.981869	0.852516	0.389804	0.090*	0.284 (8)
H47F	1.059127	0.887487	0.436844	0.090*	0.284 (8)
C48B	0.9648 (9)	0.9616 (3)	0.4077 (8)	0.049 (3)	0.284 (8)
H48D	0.971671	0.966356	0.352024	0.073*	0.284 (8)
H48E	1.022428	0.966322	0.452747	0.073*	0.284 (8)
H48F	0.920922	0.982931	0.415005	0.073*	0.284 (8)
C49B	0.8410 (6)	0.9070 (6)	0.3445 (6)	0.056 (4)	0.284 (8)
H49D	0.821845	0.875775	0.345083	0.084*	0.284 (8)
H49E	0.845064	0.914116	0.288509	0.084*	0.284 (8)
H49F	0.797498	0.927024	0.356195	0.084*	0.284 (8)
C50	0.82772 (15)	0.96168 (7)	0.65348 (14)	0.0302 (4)	
C51	0.74905 (17)	0.93991 (9)	0.67292 (17)	0.0415 (6)	
H51A	0.707962	0.926466	0.620829	0.062*	
H51B	0.717428	0.962812	0.694078	0.062*	
H51C	0.771552	0.916550	0.716264	0.062*	
C52	0.78897 (16)	1.00097 (8)	0.59236 (17)	0.0397 (6)	
H52A	0.745544	0.989460	0.539595	0.060*	
H52B	0.837358	1.016372	0.579297	0.060*	
H52C	0.759325	1.022123	0.619244	0.060*	
C53	0.89511 (18)	0.98139 (9)	0.73532 (16)	0.0434 (6)	
H53A	0.945630	0.994493	0.722265	0.065*	
H53B	0.916729	0.957504	0.777697	0.065*	
H53C	0.865827	1.004723	0.758047	0.065*	
C54	0.71609 (11)	0.71773 (6)	0.43015 (12)	0.0199 (3)	
H54A	0.651256	0.724189	0.411538	0.024*	
H54B	0.733107	0.714500	0.378453	0.024*	
C55	0.73381 (11)	0.67400 (6)	0.47843 (11)	0.0181 (3)	
C56	0.81404 (11)	0.65147 (6)	0.49064 (11)	0.0190 (3)	
H56	0.853784	0.662458	0.463438	0.023*	0.857 (4)
C57	0.83762 (12)	0.61328 (6)	0.54161 (11)	0.0192 (3)	
C58	0.77628 (13)	0.59762 (6)	0.57860 (12)	0.0223 (4)	
H58	0.791871	0.571904	0.614255	0.027*	
C59	0.69358 (12)	0.61752 (7)	0.56624 (12)	0.0219 (4)	
C60	0.67343 (11)	0.65639 (7)	0.51527 (12)	0.0206 (3)	
H60	0.617740	0.671004	0.505629	0.025*	0.143 (4)
C61	0.92834 (13)	0.59069 (7)	0.55567 (13)	0.0244 (4)	
C62	0.94060 (16)	0.54802 (8)	0.60966 (15)	0.0349 (5)	
H62A	0.892231	0.526887	0.582237	0.052*	
H62B	0.939272	0.555719	0.666405	0.052*	
H62C	0.998155	0.534161	0.614894	0.052*	
C63	0.93679 (14)	0.57830 (8)	0.46884 (14)	0.0304 (4)	
H63A	0.887427	0.558430	0.437847	0.046*	
H63B	0.993754	0.562897	0.477896	0.046*	
H63C	0.934557	0.605684	0.435584	0.046*	
C64	1.00323 (13)	0.62374 (8)	0.60294 (15)	0.0335 (5)	
H64A	1.061471	0.609641	0.612221	0.050*	
H64B	0.997492	0.631604	0.657991	0.050*	
H64C	0.998313	0.650978	0.568673	0.050*	
C65	0.62817 (14)	0.59830 (8)	0.60747 (15)	0.0327 (5)	
C66	0.61091 (16)	0.63295 (9)	0.66877 (16)	0.0403 (6)	
H66A	0.585029	0.660190	0.637104	0.060*	
H66B	0.667416	0.640399	0.713348	0.060*	
H66C	0.569293	0.620302	0.694870	0.060*	
C67	0.54135 (17)	0.58412 (11)	0.5376 (2)	0.0559 (8)	
H67A	0.513127	0.610499	0.504278	0.084*	
H67B	0.500538	0.570766	0.563928	0.084*	
H67C	0.555000	0.562032	0.500144	0.084*	
C68	0.6653 (2)	0.55572 (9)	0.66041 (19)	0.0482 (7)	
H68A	0.677181	0.532695	0.623551	0.072*	
H68B	0.621436	0.544351	0.685028	0.072*	
H68C	0.720940	0.563107	0.706248	0.072*	
O5	0.3231 (2)	0.70311 (9)	0.50018 (17)	0.0253 (7)	0.685 (8)
H5	0.347038	0.728568	0.510765	0.038*	0.685 (8)
C69	0.3784 (3)	0.67414 (13)	0.4738 (3)	0.0462 (12)	0.685 (8)
H69A	0.387186	0.686296	0.422488	0.069*	0.685 (8)
H69B	0.350314	0.644488	0.461162	0.069*	0.685 (8)
H69C	0.436337	0.671417	0.519210	0.069*	0.685 (8)
O5B	0.3750 (8)	0.6952 (2)	0.5134 (4)	0.042 (2)	0.315 (8)
H5C	0.380 (7)	0.7231 (9)	0.520 (6)	0.063*	0.315 (8)
H5D	0.341 (7)	0.690 (3)	0.462 (3)	0.063*	0.315 (8)
O6	1.02032 (15)	0.66923 (8)	0.36487 (12)	0.0432 (6)	0.843 (4)
H6	1.001717	0.691429	0.385152	0.065*	0.843 (4)
C70	1.1050 (3)	0.65658 (16)	0.4205 (2)	0.0439 (9)	0.843 (4)
H70A	1.149634	0.678422	0.416707	0.066*	0.843 (4)
H70B	1.120249	0.626776	0.404579	0.066*	0.843 (4)
H70C	1.104027	0.655737	0.478980	0.066*	0.843 (4)
O6B	1.1055 (10)	0.7095 (5)	0.3721 (9)	0.063 (4)	0.157 (4)
H6B	1.082514	0.725418	0.400806	0.095*	0.157 (4)
C70B	1.104 (3)	0.6652 (8)	0.3948 (17)	0.065 (6)	0.157 (4)
H70D	1.116875	0.663148	0.456460	0.097*	0.157 (4)
H70E	1.148844	0.648392	0.378888	0.097*	0.157 (4)
H70F	1.044521	0.652638	0.365208	0.097*	0.157 (4)
N1	0.24699 (9)	0.82353 (5)	0.38574 (9)	0.0144 (3)	
N2	0.30708 (9)	0.78810 (5)	0.25896 (9)	0.0153 (3)	
N3	0.91741 (10)	0.77394 (5)	0.62590 (9)	0.0177 (3)	
N4	0.76653 (9)	0.75710 (5)	0.48193 (9)	0.0172 (3)	
O1	0.40507 (8)	0.78699 (4)	0.51791 (7)	0.0172 (2)	
O2	0.26805 (12)	0.86608 (5)	0.07650 (10)	0.0330 (4)	0.917 (3)
H2	0.290674	0.844771	0.057669	0.049*	0.917 (3)
O2B	0.5034 (9)	0.8514 (5)	0.3518 (8)	0.0201 (3)	0.083 (3)
H2C	0.486603	0.824743	0.353287	0.030*	0.083 (3)
O3	0.59302 (10)	0.67610 (6)	0.50671 (11)	0.0295 (5)	0.857 (4)
H3	0.575190	0.690570	0.460818	0.044*	0.857 (4)
O3B	0.8753 (6)	0.6616 (3)	0.4513 (7)	0.024 (3)	0.143 (4)
H3D	0.871900	0.689051	0.438845	0.036*	0.143 (4)
O4	0.90424 (13)	0.88163 (6)	0.73783 (10)	0.0337 (5)	0.899 (4)
H4	0.943603	0.863677	0.766568	0.051*	0.899 (4)
O4B	1.0122 (8)	0.8408 (4)	0.5013 (7)	0.022 (4)	0.101 (4)
H4D	1.041665	0.820583	0.533947	0.033*	0.101 (4)
Pd1	0.36273 (2)	0.79241 (2)	0.39043 (2)	0.01248 (3)	
Pd2	0.90248 (2)	0.75700 (2)	0.50140 (2)	0.01722 (3)	
Cl1	0.49514 (3)	0.75869 (2)	0.39289 (3)	0.02383 (9)	
Cl2	1.05553 (3)	0.75220 (2)	0.52937 (3)	0.02905 (11)	
Cl3	0.87855 (4)	0.74325 (2)	0.35902 (3)	0.03647 (13)	

### Atomic displacement parameters (Å^2^)

**Table:**

	*U*^11^	*U*^22^	*U*^33^	*U*^12^	*U*^13^	*U*^23^
C1	0.0134 (7)	0.0250 (9)	0.0175 (8)	−0.0010 (7)	0.0021 (6)	−0.0029 (7)
C2	0.0145 (7)	0.0250 (9)	0.0142 (8)	0.0016 (7)	0.0021 (6)	−0.0014 (7)
C3	0.0197 (8)	0.0139 (8)	0.0249 (9)	0.0018 (7)	0.0096 (7)	0.0010 (6)
C4	0.0309 (10)	0.0181 (9)	0.0260 (10)	−0.0027 (8)	0.0089 (8)	−0.0083 (7)
C5	0.0146 (7)	0.0226 (8)	0.0172 (8)	−0.0009 (7)	0.0077 (6)	0.0005 (6)
C6	0.0163 (7)	0.0203 (8)	0.0161 (8)	0.0004 (7)	0.0069 (6)	0.0010 (6)
C7	0.0205 (8)	0.0225 (9)	0.0211 (8)	0.0021 (7)	0.0111 (7)	0.0003 (7)
C8	0.0271 (9)	0.0227 (9)	0.0182 (8)	0.0001 (7)	0.0125 (7)	−0.0017 (7)
C9	0.0240 (8)	0.0212 (8)	0.0138 (8)	−0.0039 (7)	0.0072 (6)	−0.0014 (6)
C10	0.0188 (7)	0.0206 (8)	0.0141 (8)	−0.0004 (7)	0.0062 (6)	0.0018 (6)
C11	0.0192 (7)	0.0180 (8)	0.0143 (7)	0.0009 (7)	0.0081 (6)	0.0019 (6)
C12	0.0350 (10)	0.0260 (10)	0.0278 (10)	0.0001 (9)	0.0177 (9)	−0.0087 (8)
C13	0.0653 (18)	0.0386 (14)	0.0454 (15)	0.0174 (13)	0.0202 (13)	−0.0054 (12)
C14	0.0624 (17)	0.0483 (16)	0.0654 (18)	−0.0167 (14)	0.0506 (15)	−0.0241 (14)
C15	0.0536 (14)	0.0372 (13)	0.0344 (12)	−0.0036 (11)	0.0212 (11)	−0.0139 (10)
C16	0.0166 (7)	0.0236 (9)	0.0152 (8)	−0.0016 (7)	0.0035 (6)	0.0024 (6)
C17	0.0196 (8)	0.0282 (10)	0.0242 (9)	−0.0026 (8)	0.0082 (7)	0.0057 (8)
C18	0.0211 (8)	0.0255 (9)	0.0235 (9)	0.0006 (8)	0.0052 (7)	0.0051 (7)
C19	0.0241 (9)	0.0357 (11)	0.0210 (9)	−0.0026 (8)	−0.0002 (7)	−0.0032 (8)
C20	0.0224 (8)	0.0240 (9)	0.0139 (8)	0.0036 (7)	0.0090 (6)	−0.0010 (6)
C21	0.0202 (8)	0.0204 (8)	0.0158 (8)	0.0021 (7)	0.0095 (6)	0.0029 (6)
C22	0.0193 (8)	0.0277 (9)	0.0151 (8)	0.0027 (7)	0.0079 (6)	0.0033 (7)
C23	0.0213 (8)	0.0276 (10)	0.0185 (8)	−0.0027 (7)	0.0099 (7)	0.0000 (7)
C24	0.0246 (9)	0.0232 (9)	0.0227 (9)	0.0002 (7)	0.0125 (7)	0.0018 (7)
C25	0.0217 (8)	0.0244 (9)	0.0194 (8)	0.0047 (7)	0.0104 (7)	0.0042 (7)
C26	0.0218 (8)	0.0266 (9)	0.0155 (8)	0.0042 (7)	0.0065 (7)	0.0019 (7)
C27	0.0283 (9)	0.0310 (11)	0.0220 (9)	−0.0034 (8)	0.0072 (8)	−0.0010 (8)
C28	0.0636 (17)	0.0521 (16)	0.0329 (13)	−0.0340 (14)	0.0032 (12)	0.0030 (12)
C29	0.0257 (10)	0.0689 (19)	0.0408 (14)	0.0030 (12)	0.0016 (10)	−0.0218 (13)
C30	0.0417 (12)	0.0330 (11)	0.0198 (9)	0.0031 (10)	0.0085 (9)	−0.0029 (8)
C31	0.0241 (9)	0.0231 (9)	0.0247 (9)	0.0063 (8)	0.0102 (7)	0.0061 (7)
C32	0.0258 (9)	0.0290 (11)	0.0387 (12)	0.0067 (8)	0.0113 (9)	0.0069 (9)
C33	0.0408 (12)	0.0397 (12)	0.0238 (10)	0.0187 (10)	0.0129 (9)	0.0132 (9)
C34	0.0360 (11)	0.0259 (10)	0.0376 (12)	0.0029 (9)	0.0109 (9)	0.0079 (9)
C35	0.0272 (9)	0.0251 (9)	0.0226 (9)	0.0019 (8)	0.0172 (7)	0.0005 (7)
C36	0.0222 (8)	0.0219 (9)	0.0272 (9)	0.0018 (7)	0.0176 (7)	0.0017 (7)
C37	0.0385 (11)	0.0247 (10)	0.0163 (9)	0.0086 (8)	0.0115 (8)	0.0048 (7)
C38	0.0190 (8)	0.0242 (10)	0.0375 (11)	0.0018 (7)	0.0065 (8)	0.0133 (8)
C39	0.0252 (9)	0.0256 (9)	0.0168 (8)	0.0005 (8)	0.0005 (7)	−0.0001 (7)
C40	0.0208 (8)	0.0230 (9)	0.0192 (9)	−0.0031 (7)	0.0015 (7)	−0.0006 (7)
C41	0.0182 (8)	0.0226 (9)	0.0228 (9)	0.0006 (7)	0.0055 (7)	−0.0012 (7)
C42	0.0178 (8)	0.0222 (9)	0.0217 (9)	−0.0028 (7)	0.0063 (7)	−0.0009 (7)
C43	0.0195 (8)	0.0187 (8)	0.0262 (9)	−0.0032 (7)	0.0056 (7)	−0.0029 (7)
C44	0.0221 (8)	0.0213 (9)	0.0248 (9)	−0.0063 (7)	0.0069 (7)	−0.0085 (7)
C45	0.0270 (9)	0.0259 (10)	0.0189 (9)	−0.0061 (8)	0.0047 (7)	−0.0055 (7)
C46	0.0223 (9)	0.0295 (12)	0.0238 (19)	0.0036 (9)	0.0115 (9)	0.0053 (10)
C47	0.030 (2)	0.177 (10)	0.052 (4)	0.025 (4)	0.026 (2)	0.043 (5)
C48	0.076 (3)	0.0276 (17)	0.037 (2)	0.0010 (19)	0.025 (2)	0.0098 (14)
C49	0.065 (3)	0.041 (2)	0.0230 (16)	−0.007 (2)	0.0152 (16)	−0.0002 (15)
C46B	0.0223 (9)	0.0295 (12)	0.0238 (19)	0.0036 (9)	0.0115 (9)	0.0053 (10)
C47B	0.073 (11)	0.086 (10)	0.033 (6)	0.066 (9)	0.035 (8)	0.035 (7)
C48B	0.070 (8)	0.035 (5)	0.059 (7)	−0.011 (5)	0.045 (6)	0.005 (4)
C49B	0.035 (5)	0.114 (12)	0.020 (4)	−0.021 (6)	0.010 (4)	0.007 (5)
C50	0.0325 (10)	0.0257 (10)	0.0349 (11)	−0.0077 (9)	0.0146 (9)	−0.0142 (9)
C51	0.0402 (12)	0.0415 (14)	0.0515 (15)	−0.0076 (11)	0.0270 (12)	−0.0154 (11)
C52	0.0388 (12)	0.0252 (11)	0.0575 (16)	0.0020 (10)	0.0193 (11)	−0.0127 (10)
C53	0.0499 (14)	0.0412 (14)	0.0401 (14)	−0.0137 (12)	0.0164 (11)	−0.0247 (11)
C54	0.0139 (7)	0.0249 (9)	0.0192 (8)	0.0001 (7)	0.0032 (6)	0.0056 (7)
C55	0.0139 (7)	0.0220 (8)	0.0165 (8)	−0.0012 (7)	0.0027 (6)	0.0019 (6)
C56	0.0166 (7)	0.0227 (9)	0.0169 (8)	−0.0015 (7)	0.0043 (6)	−0.0008 (7)
C57	0.0185 (8)	0.0197 (8)	0.0164 (8)	0.0005 (7)	0.0019 (6)	−0.0023 (6)
C58	0.0257 (9)	0.0195 (8)	0.0196 (9)	−0.0019 (7)	0.0049 (7)	0.0011 (7)
C59	0.0195 (8)	0.0242 (9)	0.0212 (9)	−0.0050 (7)	0.0057 (7)	0.0004 (7)
C60	0.0137 (7)	0.0262 (9)	0.0198 (8)	−0.0008 (7)	0.0029 (6)	0.0022 (7)
C61	0.0218 (8)	0.0233 (9)	0.0247 (9)	0.0042 (7)	0.0035 (7)	−0.0028 (7)
C62	0.0362 (11)	0.0291 (11)	0.0325 (12)	0.0114 (9)	0.0025 (9)	0.0028 (9)
C63	0.0256 (9)	0.0338 (11)	0.0305 (11)	0.0042 (9)	0.0078 (8)	−0.0080 (9)
C64	0.0188 (9)	0.0328 (11)	0.0414 (13)	0.0047 (8)	0.0002 (8)	−0.0139 (9)
C65	0.0266 (10)	0.0385 (12)	0.0348 (11)	−0.0073 (9)	0.0127 (9)	0.0074 (9)
C66	0.0344 (11)	0.0534 (15)	0.0414 (13)	0.0084 (11)	0.0236 (10)	0.0136 (11)
C67	0.0356 (13)	0.068 (2)	0.0628 (19)	−0.0263 (14)	0.0140 (13)	0.0035 (15)
C68	0.0594 (16)	0.0381 (14)	0.0593 (17)	−0.0032 (13)	0.0362 (14)	0.0174 (12)
O5	0.0307 (15)	0.0231 (12)	0.0259 (12)	−0.0020 (11)	0.0144 (11)	−0.0014 (9)
C69	0.055 (2)	0.0218 (17)	0.081 (3)	−0.0019 (16)	0.048 (2)	−0.003 (2)
O5B	0.076 (6)	0.021 (3)	0.038 (3)	−0.006 (3)	0.030 (4)	0.003 (2)
O6	0.0493 (13)	0.0515 (14)	0.0208 (10)	0.0174 (10)	0.0011 (9)	−0.0052 (8)
C70	0.0377 (15)	0.064 (2)	0.033 (2)	0.0077 (17)	0.0156 (16)	−0.0036 (16)
O6B	0.079 (8)	0.072 (8)	0.053 (7)	−0.012 (7)	0.041 (6)	−0.011 (6)
C70B	0.078 (10)	0.083 (10)	0.039 (10)	0.007 (9)	0.027 (9)	−0.005 (9)
N1	0.0122 (6)	0.0162 (7)	0.0139 (6)	0.0005 (5)	0.0033 (5)	−0.0014 (5)
N2	0.0167 (6)	0.0152 (7)	0.0141 (6)	0.0005 (5)	0.0051 (5)	−0.0017 (5)
N3	0.0210 (7)	0.0190 (7)	0.0146 (7)	0.0020 (6)	0.0077 (6)	0.0015 (5)
N4	0.0145 (6)	0.0208 (7)	0.0171 (7)	0.0022 (6)	0.0063 (5)	0.0058 (6)
O1	0.0178 (5)	0.0212 (6)	0.0125 (5)	0.0049 (5)	0.0051 (4)	−0.0003 (5)
O2	0.0432 (10)	0.0242 (8)	0.0200 (8)	0.0074 (7)	−0.0048 (7)	−0.0029 (6)
O2B	0.0193 (8)	0.0277 (9)	0.0151 (8)	0.0027 (7)	0.0079 (6)	0.0033 (7)
O3	0.0151 (7)	0.0379 (10)	0.0363 (10)	0.0050 (7)	0.0096 (7)	0.0192 (8)
O3B	0.018 (4)	0.024 (5)	0.038 (6)	−0.002 (4)	0.019 (4)	0.001 (4)
O4	0.0495 (11)	0.0335 (10)	0.0188 (8)	0.0011 (8)	0.0122 (7)	−0.0041 (7)
O4B	0.020 (6)	0.032 (8)	0.014 (6)	0.014 (5)	0.008 (5)	0.005 (5)
Pd1	0.01173 (5)	0.01425 (6)	0.01174 (6)	0.00192 (5)	0.00432 (4)	−0.00027 (4)
Pd2	0.01268 (6)	0.02854 (7)	0.01189 (6)	0.00021 (5)	0.00602 (4)	0.00231 (5)
Cl1	0.02027 (19)	0.0316 (2)	0.0220 (2)	0.01215 (18)	0.01026 (16)	0.00532 (18)
Cl2	0.01481 (18)	0.0442 (3)	0.0303 (2)	0.00095 (19)	0.01044 (17)	0.0005 (2)
Cl3	0.0303 (2)	0.0669 (4)	0.0157 (2)	−0.0060 (3)	0.01236 (19)	−0.0033 (2)

### Geometric parameters (Å, º)

**Table:**

C1—N1	1.496 (2)	C41—H41	0.9500
C1—C2	1.518 (2)	C42—C43	1.398 (3)
C1—H1A	0.9900	C42—C46B	1.46 (3)
C1—H1B	0.9900	C42—C46	1.562 (10)
C2—N2	1.503 (2)	C43—C44	1.395 (3)
C2—H2A	0.9900	C43—H43	0.9500
C2—H2B	0.9900	C44—C45	1.410 (3)
C3—N1	1.488 (2)	C44—C50	1.538 (3)
C3—H3A	0.9800	C45—O4	1.382 (2)
C3—H3B	0.9800	C45—H45	0.9500
C3—H3C	0.9800	C46—C47	1.518 (7)
C4—N2	1.498 (2)	C46—C48	1.530 (7)
C4—H4A	0.9800	C46—C49	1.533 (7)
C4—H4B	0.9800	C47—H47A	0.9800
C4—H4C	0.9800	C47—H47B	0.9800
C5—C6	1.505 (2)	C47—H47C	0.9800
C5—N1	1.509 (2)	C48—H48A	0.9800
C5—H5A	0.9900	C48—H48B	0.9800
C5—H5B	0.9900	C48—H48C	0.9800
C6—C7	1.393 (2)	C49—H49A	0.9800
C6—C11	1.417 (2)	C49—H49B	0.9800
C7—C8	1.399 (3)	C49—H49C	0.9800
C7—H7	0.9500	C46B—C47B	1.519 (16)
C8—C9	1.396 (3)	C46B—C49B	1.531 (16)
C8—C12	1.534 (3)	C46B—C48B	1.534 (15)
C9—C10	1.399 (2)	C47B—H47D	0.9800
C9—H9	0.9500	C47B—H47E	0.9800
C10—C11	1.419 (2)	C47B—H47F	0.9800
C10—C16	1.542 (2)	C48B—H48D	0.9800
C11—O1	1.346 (2)	C48B—H48E	0.9800
C12—C14	1.526 (3)	C48B—H48F	0.9800
C12—C15	1.531 (3)	C49B—H49D	0.9800
C12—C13	1.538 (3)	C49B—H49E	0.9800
C13—H13A	0.9800	C49B—H49F	0.9800
C13—H13B	0.9800	C50—C51	1.535 (3)
C13—H13C	0.9800	C50—C52	1.538 (3)
C14—H14A	0.9800	C50—C53	1.540 (3)
C14—H14B	0.9800	C51—H51A	0.9800
C14—H14C	0.9800	C51—H51B	0.9800
C15—H15A	0.9800	C51—H51C	0.9800
C15—H15B	0.9800	C52—H52A	0.9800
C15—H15C	0.9800	C52—H52B	0.9800
C16—C19	1.535 (3)	C52—H52C	0.9800
C16—C18	1.539 (3)	C53—H53A	0.9800
C16—C17	1.542 (3)	C53—H53B	0.9800
C17—H17A	0.9800	C53—H53C	0.9800
C17—H17B	0.9800	C54—C55	1.507 (2)
C17—H17C	0.9800	C54—N4	1.516 (2)
C18—H18A	0.9800	C54—H54A	0.9900
C18—H18B	0.9800	C54—H54B	0.9900
C18—H18C	0.9800	C55—C56	1.394 (2)
C19—H19A	0.9800	C55—C60	1.402 (2)
C19—H19B	0.9800	C56—O3B	1.377 (9)
C19—H19C	0.9800	C56—C57	1.394 (3)
C20—C21	1.509 (3)	C56—H56	0.9500
C20—N2	1.511 (2)	C57—C58	1.396 (3)
C20—H20A	0.9900	C57—C61	1.536 (3)
C20—H20B	0.9900	C58—C59	1.392 (3)
C21—C22	1.396 (3)	C58—H58	0.9500
C21—C26	1.404 (2)	C59—C60	1.408 (3)
C22—O2B	1.350 (10)	C59—C65	1.535 (3)
C22—C23	1.392 (3)	C60—O3	1.369 (2)
C22—H22	0.9500	C60—H60	0.9500
C23—C24	1.397 (3)	C61—C62	1.531 (3)
C23—C27	1.537 (3)	C61—C64	1.542 (3)
C24—C25	1.396 (3)	C61—C63	1.542 (3)
C24—H24	0.9500	C62—H62A	0.9800
C25—C26	1.406 (3)	C62—H62B	0.9800
C25—C31	1.543 (3)	C62—H62C	0.9800
C26—O2	1.383 (2)	C63—H63A	0.9800
C26—H26	0.9500	C63—H63B	0.9800
C27—C28	1.532 (3)	C63—H63C	0.9800
C27—C30	1.533 (3)	C64—H64A	0.9800
C27—C29	1.539 (3)	C64—H64B	0.9800
C28—H28A	0.9800	C64—H64C	0.9800
C28—H28B	0.9800	C65—C67	1.536 (3)
C28—H28C	0.9800	C65—C66	1.540 (4)
C29—H29A	0.9800	C65—C68	1.545 (3)
C29—H29B	0.9800	C66—H66A	0.9800
C29—H29C	0.9800	C66—H66B	0.9800
C30—H30A	0.9800	C66—H66C	0.9800
C30—H30B	0.9800	C67—H67A	0.9800
C30—H30C	0.9800	C67—H67B	0.9800
C31—C33	1.532 (3)	C67—H67C	0.9800
C31—C34	1.535 (3)	C68—H68A	0.9800
C31—C32	1.537 (3)	C68—H68B	0.9800
C32—H32A	0.9800	C68—H68C	0.9800
C32—H32B	0.9800	O5—C69	1.403 (5)
C32—H32C	0.9800	O5—H5	0.8400
C33—H33A	0.9800	C69—H69A	0.9800
C33—H33B	0.9800	C69—H69B	0.9800
C33—H33C	0.9800	C69—H69C	0.9800
C34—H34A	0.9800	O5B—H5C	0.84 (2)
C34—H34B	0.9800	O5B—H5D	0.86 (2)
C34—H34C	0.9800	O6—C70	1.405 (5)
C35—C36	1.503 (3)	O6—H6	0.8400
C35—N3	1.503 (2)	C70—H70A	0.9800
C35—H35A	0.9900	C70—H70B	0.9800
C35—H35B	0.9900	C70—H70C	0.9800
C36—N4	1.498 (2)	O6B—C70B	1.373 (18)
C36—H36A	0.9900	O6B—H6B	0.8400
C36—H36B	0.9900	C70B—H70D	0.9800
C37—N3	1.490 (2)	C70B—H70E	0.9800
C37—H37A	0.9800	C70B—H70F	0.9800
C37—H37B	0.9800	N1—Pd1	2.0369 (14)
C37—H37C	0.9800	N2—Pd1	2.0739 (14)
C38—N4	1.496 (2)	N3—Pd2	2.0699 (15)
C38—H38A	0.9800	N4—Pd2	2.0735 (14)
C38—H38B	0.9800	O1—Pd1	2.0085 (12)
C38—H38C	0.9800	O2—H2	0.8400
C39—C40	1.511 (3)	O2B—H2C	0.8400
C39—N3	1.515 (2)	O3—H3	0.8400
C39—H39A	0.9900	O3B—H3D	0.8400
C39—H39B	0.9900	O4—H4	0.8400
C40—C41	1.396 (3)	O4B—H4D	0.8400
C40—C45	1.403 (3)	Pd1—Cl1	2.3186 (4)
C41—C42	1.393 (3)	Pd2—Cl3	2.3102 (5)
C41—O4B	1.410 (9)	Pd2—Cl2	2.3216 (5)
			
N1—C1—C2	109.06 (14)	C45—C44—C50	121.37 (18)
N1—C1—H1A	109.9	O4—C45—C40	122.71 (19)
C2—C1—H1A	109.9	O4—C45—C44	116.44 (18)
N1—C1—H1B	109.9	C40—C45—C44	120.79 (18)
C2—C1—H1B	109.9	C40—C45—H45	119.6
H1A—C1—H1B	108.3	C44—C45—H45	119.6
N2—C2—C1	108.93 (14)	C47—C46—C48	109.4 (6)
N2—C2—H2A	109.9	C47—C46—C49	109.2 (6)
C1—C2—H2A	109.9	C48—C46—C49	107.5 (5)
N2—C2—H2B	109.9	C47—C46—C42	109.8 (6)
C1—C2—H2B	109.9	C48—C46—C42	111.7 (5)
H2A—C2—H2B	108.3	C49—C46—C42	109.2 (5)
N1—C3—H3A	109.5	C46—C47—H47A	109.5
N1—C3—H3B	109.5	C46—C47—H47B	109.5
H3A—C3—H3B	109.5	H47A—C47—H47B	109.5
N1—C3—H3C	109.5	C46—C47—H47C	109.5
H3A—C3—H3C	109.5	H47A—C47—H47C	109.5
H3B—C3—H3C	109.5	H47B—C47—H47C	109.5
N2—C4—H4A	109.5	C46—C48—H48A	109.5
N2—C4—H4B	109.5	C46—C48—H48B	109.5
H4A—C4—H4B	109.5	H48A—C48—H48B	109.5
N2—C4—H4C	109.5	C46—C48—H48C	109.5
H4A—C4—H4C	109.5	H48A—C48—H48C	109.5
H4B—C4—H4C	109.5	H48B—C48—H48C	109.5
C6—C5—N1	111.11 (14)	C46—C49—H49A	109.5
C6—C5—H5A	109.4	C46—C49—H49B	109.5
N1—C5—H5A	109.4	H49A—C49—H49B	109.5
C6—C5—H5B	109.4	C46—C49—H49C	109.5
N1—C5—H5B	109.4	H49A—C49—H49C	109.5
H5A—C5—H5B	108.0	H49B—C49—H49C	109.5
C7—C6—C11	120.74 (16)	C42—C46B—C47B	112.4 (14)
C7—C6—C5	119.95 (15)	C42—C46B—C49B	109.6 (13)
C11—C6—C5	119.27 (15)	C47B—C46B—C49B	109.3 (15)
C6—C7—C8	121.32 (17)	C42—C46B—C48B	111.8 (14)
C6—C7—H7	119.3	C47B—C46B—C48B	104.6 (14)
C8—C7—H7	119.3	C49B—C46B—C48B	109.1 (14)
C9—C8—C7	117.04 (16)	C46B—C47B—H47D	109.5
C9—C8—C12	122.84 (17)	C46B—C47B—H47E	109.5
C7—C8—C12	120.11 (17)	H47D—C47B—H47E	109.5
C8—C9—C10	124.11 (17)	C46B—C47B—H47F	109.5
C8—C9—H9	117.9	H47D—C47B—H47F	109.5
C10—C9—H9	117.9	H47E—C47B—H47F	109.5
C9—C10—C11	117.78 (16)	C46B—C48B—H48D	109.5
C9—C10—C16	120.90 (16)	C46B—C48B—H48E	109.5
C11—C10—C16	121.31 (15)	H48D—C48B—H48E	109.5
O1—C11—C6	120.19 (15)	C46B—C48B—H48F	109.5
O1—C11—C10	120.80 (15)	H48D—C48B—H48F	109.5
C6—C11—C10	119.00 (16)	H48E—C48B—H48F	109.5
C14—C12—C15	109.5 (2)	C46B—C49B—H49D	109.5
C14—C12—C8	109.80 (18)	C46B—C49B—H49E	109.5
C15—C12—C8	112.38 (18)	H49D—C49B—H49E	109.5
C14—C12—C13	108.8 (2)	C46B—C49B—H49F	109.5
C15—C12—C13	106.8 (2)	H49D—C49B—H49F	109.5
C8—C12—C13	109.47 (18)	H49E—C49B—H49F	109.5
C12—C13—H13A	109.5	C51—C50—C44	110.77 (17)
C12—C13—H13B	109.5	C51—C50—C52	106.7 (2)
H13A—C13—H13B	109.5	C44—C50—C52	111.90 (19)
C12—C13—H13C	109.5	C51—C50—C53	110.3 (2)
H13A—C13—H13C	109.5	C44—C50—C53	109.49 (18)
H13B—C13—H13C	109.5	C52—C50—C53	107.55 (19)
C12—C14—H14A	109.5	C50—C51—H51A	109.5
C12—C14—H14B	109.5	C50—C51—H51B	109.5
H14A—C14—H14B	109.5	H51A—C51—H51B	109.5
C12—C14—H14C	109.5	C50—C51—H51C	109.5
H14A—C14—H14C	109.5	H51A—C51—H51C	109.5
H14B—C14—H14C	109.5	H51B—C51—H51C	109.5
C12—C15—H15A	109.5	C50—C52—H52A	109.5
C12—C15—H15B	109.5	C50—C52—H52B	109.5
H15A—C15—H15B	109.5	H52A—C52—H52B	109.5
C12—C15—H15C	109.5	C50—C52—H52C	109.5
H15A—C15—H15C	109.5	H52A—C52—H52C	109.5
H15B—C15—H15C	109.5	H52B—C52—H52C	109.5
C19—C16—C18	107.87 (15)	C50—C53—H53A	109.5
C19—C16—C17	107.20 (16)	C50—C53—H53B	109.5
C18—C16—C17	109.50 (16)	H53A—C53—H53B	109.5
C19—C16—C10	112.13 (16)	C50—C53—H53C	109.5
C18—C16—C10	110.82 (15)	H53A—C53—H53C	109.5
C17—C16—C10	109.22 (14)	H53B—C53—H53C	109.5
C16—C17—H17A	109.5	C55—C54—N4	113.32 (14)
C16—C17—H17B	109.5	C55—C54—H54A	108.9
H17A—C17—H17B	109.5	N4—C54—H54A	108.9
C16—C17—H17C	109.5	C55—C54—H54B	108.9
H17A—C17—H17C	109.5	N4—C54—H54B	108.9
H17B—C17—H17C	109.5	H54A—C54—H54B	107.7
C16—C18—H18A	109.5	C56—C55—C60	118.83 (17)
C16—C18—H18B	109.5	C56—C55—C54	119.49 (16)
H18A—C18—H18B	109.5	C60—C55—C54	121.60 (16)
C16—C18—H18C	109.5	O3B—C56—C55	125.6 (4)
H18A—C18—H18C	109.5	O3B—C56—C57	112.4 (4)
H18B—C18—H18C	109.5	C55—C56—C57	121.86 (17)
C16—C19—H19A	109.5	C55—C56—H56	119.1
C16—C19—H19B	109.5	C57—C56—H56	119.1
H19A—C19—H19B	109.5	C56—C57—C58	117.18 (17)
C16—C19—H19C	109.5	C56—C57—C61	119.84 (17)
H19A—C19—H19C	109.5	C58—C57—C61	122.97 (17)
H19B—C19—H19C	109.5	C59—C58—C57	123.78 (18)
C21—C20—N2	114.07 (14)	C59—C58—H58	118.1
C21—C20—H20A	108.7	C57—C58—H58	118.1
N2—C20—H20A	108.7	C58—C59—C60	116.87 (17)
C21—C20—H20B	108.7	C58—C59—C65	121.44 (18)
N2—C20—H20B	108.7	C60—C59—C65	121.68 (17)
H20A—C20—H20B	107.6	O3—C60—C55	123.03 (17)
C22—C21—C26	119.40 (17)	O3—C60—C59	115.54 (17)
C22—C21—C20	119.85 (16)	C55—C60—C59	121.40 (17)
C26—C21—C20	120.75 (16)	C55—C60—H60	119.3
O2B—C22—C23	109.9 (6)	C59—C60—H60	119.3
O2B—C22—C21	128.2 (6)	C62—C61—C57	112.43 (18)
C23—C22—C21	121.46 (17)	C62—C61—C64	107.75 (17)
C23—C22—H22	119.3	C57—C61—C64	108.80 (16)
C21—C22—H22	119.3	C62—C61—C63	108.59 (17)
C22—C23—C24	117.35 (17)	C57—C61—C63	109.50 (16)
C22—C23—C27	120.83 (17)	C64—C61—C63	109.73 (18)
C24—C23—C27	121.78 (18)	C61—C62—H62A	109.5
C25—C24—C23	123.80 (18)	C61—C62—H62B	109.5
C25—C24—H24	118.1	H62A—C62—H62B	109.5
C23—C24—H24	118.1	C61—C62—H62C	109.5
C24—C25—C26	116.97 (17)	H62A—C62—H62C	109.5
C24—C25—C31	121.19 (18)	H62B—C62—H62C	109.5
C26—C25—C31	121.79 (17)	C61—C63—H63A	109.5
O2—C26—C21	119.66 (17)	C61—C63—H63B	109.5
O2—C26—C25	119.30 (17)	H63A—C63—H63B	109.5
C21—C26—C25	120.98 (17)	C61—C63—H63C	109.5
C21—C26—H26	119.5	H63A—C63—H63C	109.5
C25—C26—H26	119.5	H63B—C63—H63C	109.5
C28—C27—C30	107.6 (2)	C61—C64—H64A	109.5
C28—C27—C23	111.93 (18)	C61—C64—H64B	109.5
C30—C27—C23	108.53 (17)	H64A—C64—H64B	109.5
C28—C27—C29	110.0 (2)	C61—C64—H64C	109.5
C30—C27—C29	108.28 (19)	H64A—C64—H64C	109.5
C23—C27—C29	110.36 (18)	H64B—C64—H64C	109.5
C27—C28—H28A	109.5	C59—C65—C67	109.51 (19)
C27—C28—H28B	109.5	C59—C65—C66	110.16 (18)
H28A—C28—H28B	109.5	C67—C65—C66	111.9 (2)
C27—C28—H28C	109.5	C59—C65—C68	112.03 (19)
H28A—C28—H28C	109.5	C67—C65—C68	106.3 (2)
H28B—C28—H28C	109.5	C66—C65—C68	106.9 (2)
C27—C29—H29A	109.5	C65—C66—H66A	109.5
C27—C29—H29B	109.5	C65—C66—H66B	109.5
H29A—C29—H29B	109.5	H66A—C66—H66B	109.5
C27—C29—H29C	109.5	C65—C66—H66C	109.5
H29A—C29—H29C	109.5	H66A—C66—H66C	109.5
H29B—C29—H29C	109.5	H66B—C66—H66C	109.5
C27—C30—H30A	109.5	C65—C67—H67A	109.5
C27—C30—H30B	109.5	C65—C67—H67B	109.5
H30A—C30—H30B	109.5	H67A—C67—H67B	109.5
C27—C30—H30C	109.5	C65—C67—H67C	109.5
H30A—C30—H30C	109.5	H67A—C67—H67C	109.5
H30B—C30—H30C	109.5	H67B—C67—H67C	109.5
C33—C31—C34	107.34 (18)	C65—C68—H68A	109.5
C33—C31—C32	109.30 (18)	C65—C68—H68B	109.5
C34—C31—C32	108.01 (17)	H68A—C68—H68B	109.5
C33—C31—C25	111.72 (16)	C65—C68—H68C	109.5
C34—C31—C25	112.10 (17)	H68A—C68—H68C	109.5
C32—C31—C25	108.29 (16)	H68B—C68—H68C	109.5
C31—C32—H32A	109.5	C69—O5—H5	109.5
C31—C32—H32B	109.5	O5—C69—H69A	109.5
H32A—C32—H32B	109.5	O5—C69—H69B	109.5
C31—C32—H32C	109.5	H69A—C69—H69B	109.5
H32A—C32—H32C	109.5	O5—C69—H69C	109.5
H32B—C32—H32C	109.5	H69A—C69—H69C	109.5
C31—C33—H33A	109.5	H69B—C69—H69C	109.5
C31—C33—H33B	109.5	H5C—O5B—H5D	107 (3)
H33A—C33—H33B	109.5	C70—O6—H6	109.5
C31—C33—H33C	109.5	O6—C70—H70A	109.5
H33A—C33—H33C	109.5	O6—C70—H70B	109.5
H33B—C33—H33C	109.5	H70A—C70—H70B	109.5
C31—C34—H34A	109.5	O6—C70—H70C	109.5
C31—C34—H34B	109.5	H70A—C70—H70C	109.5
H34A—C34—H34B	109.5	H70B—C70—H70C	109.5
C31—C34—H34C	109.5	C70B—O6B—H6B	109.5
H34A—C34—H34C	109.5	O6B—C70B—H70D	109.5
H34B—C34—H34C	109.5	O6B—C70B—H70E	109.5
C36—C35—N3	109.55 (15)	H70D—C70B—H70E	109.5
C36—C35—H35A	109.8	O6B—C70B—H70F	109.5
N3—C35—H35A	109.8	H70D—C70B—H70F	109.5
C36—C35—H35B	109.8	H70E—C70B—H70F	109.5
N3—C35—H35B	109.8	C3—N1—C1	110.84 (14)
H35A—C35—H35B	108.2	C3—N1—C5	110.38 (13)
N4—C36—C35	110.01 (14)	C1—N1—C5	109.91 (13)
N4—C36—H36A	109.7	C3—N1—Pd1	111.59 (10)
C35—C36—H36A	109.7	C1—N1—Pd1	104.61 (10)
N4—C36—H36B	109.7	C5—N1—Pd1	109.36 (10)
C35—C36—H36B	109.7	C4—N2—C2	108.85 (14)
H36A—C36—H36B	108.2	C4—N2—C20	106.92 (14)
N3—C37—H37A	109.5	C2—N2—C20	111.12 (13)
N3—C37—H37B	109.5	C4—N2—Pd1	105.61 (11)
H37A—C37—H37B	109.5	C2—N2—Pd1	107.53 (10)
N3—C37—H37C	109.5	C20—N2—Pd1	116.48 (10)
H37A—C37—H37C	109.5	C37—N3—C35	110.58 (15)
H37B—C37—H37C	109.5	C37—N3—C39	107.41 (14)
N4—C38—H38A	109.5	C35—N3—C39	110.12 (14)
N4—C38—H38B	109.5	C37—N3—Pd2	106.95 (11)
H38A—C38—H38B	109.5	C35—N3—Pd2	106.93 (11)
N4—C38—H38C	109.5	C39—N3—Pd2	114.81 (11)
H38A—C38—H38C	109.5	C38—N4—C36	110.64 (15)
H38B—C38—H38C	109.5	C38—N4—C54	107.70 (14)
C40—C39—N3	112.59 (14)	C36—N4—C54	110.40 (14)
C40—C39—H39A	109.1	C38—N4—Pd2	107.06 (11)
N3—C39—H39A	109.1	C36—N4—Pd2	106.65 (11)
C40—C39—H39B	109.1	C54—N4—Pd2	114.35 (11)
N3—C39—H39B	109.1	C11—O1—Pd1	121.65 (10)
H39A—C39—H39B	107.8	C26—O2—H2	109.5
C41—C40—C45	119.42 (18)	C22—O2B—H2C	109.5
C41—C40—C39	119.16 (17)	C60—O3—H3	109.5
C45—C40—C39	121.31 (18)	C56—O3B—H3D	109.5
C42—C41—C40	121.61 (18)	C45—O4—H4	109.5
C42—C41—O4B	110.6 (5)	C41—O4B—H4D	109.5
C40—C41—O4B	127.5 (5)	O1—Pd1—N1	93.18 (5)
C42—C41—H41	119.2	O1—Pd1—N2	170.29 (5)
C40—C41—H41	119.2	N1—Pd1—N2	85.84 (6)
C41—C42—C43	117.19 (18)	O1—Pd1—Cl1	88.04 (4)
C41—C42—C46B	122.6 (7)	N1—Pd1—Cl1	178.20 (4)
C43—C42—C46B	120.2 (7)	N2—Pd1—Cl1	93.17 (4)
C41—C42—C46	120.0 (3)	N3—Pd2—N4	85.44 (6)
C43—C42—C46	122.7 (3)	N3—Pd2—Cl3	175.32 (5)
C44—C43—C42	123.79 (18)	N4—Pd2—Cl3	91.99 (4)
C44—C43—H43	118.1	N3—Pd2—Cl2	93.02 (4)
C42—C43—H43	118.1	N4—Pd2—Cl2	175.77 (4)
C43—C44—C45	117.08 (18)	Cl3—Pd2—Cl2	89.801 (19)
C43—C44—C50	121.56 (19)		
			
N1—C1—C2—N2	53.29 (19)	C41—C40—C45—C44	4.1 (3)
N1—C5—C6—C7	118.98 (18)	C39—C40—C45—C44	−172.13 (17)
N1—C5—C6—C11	−58.7 (2)	C43—C44—C45—O4	−179.38 (17)
C11—C6—C7—C8	0.1 (3)	C50—C44—C45—O4	0.9 (3)
C5—C6—C7—C8	−177.60 (17)	C43—C44—C45—C40	−2.1 (3)
C6—C7—C8—C9	0.1 (3)	C50—C44—C45—C40	178.18 (17)
C6—C7—C8—C12	178.80 (18)	C41—C42—C46—C47	−50.7 (6)
C7—C8—C9—C10	0.3 (3)	C43—C42—C46—C47	132.9 (5)
C12—C8—C9—C10	−178.30 (18)	C41—C42—C46—C48	−172.2 (3)
C8—C9—C10—C11	−0.9 (3)	C43—C42—C46—C48	11.3 (6)
C8—C9—C10—C16	177.85 (17)	C41—C42—C46—C49	69.0 (5)
C7—C6—C11—O1	178.73 (16)	C43—C42—C46—C49	−107.5 (4)
C5—C6—C11—O1	−3.6 (2)	C41—C42—C46B—C47B	−14.7 (15)
C7—C6—C11—C10	−0.7 (3)	C43—C42—C46B—C47B	165.7 (10)
C5—C6—C11—C10	176.96 (16)	C41—C42—C46B—C49B	107.0 (11)
C9—C10—C11—O1	−178.33 (16)	C43—C42—C46B—C49B	−72.6 (12)
C16—C10—C11—O1	2.9 (3)	C41—C42—C46B—C48B	−131.9 (10)
C9—C10—C11—C6	1.1 (2)	C43—C42—C46B—C48B	48.5 (13)
C16—C10—C11—C6	−177.67 (16)	C43—C44—C50—C51	121.2 (2)
C9—C8—C12—C14	−118.9 (2)	C45—C44—C50—C51	−59.1 (3)
C7—C8—C12—C14	62.5 (3)	C43—C44—C50—C52	2.3 (3)
C9—C8—C12—C15	3.3 (3)	C45—C44—C50—C52	−178.01 (18)
C7—C8—C12—C15	−175.29 (19)	C43—C44—C50—C53	−116.9 (2)
C9—C8—C12—C13	121.8 (2)	C45—C44—C50—C53	62.8 (3)
C7—C8—C12—C13	−56.8 (3)	N4—C54—C55—C56	−76.3 (2)
C9—C10—C16—C19	−0.2 (2)	N4—C54—C55—C60	100.40 (19)
C11—C10—C16—C19	178.51 (17)	C60—C55—C56—O3B	172.4 (6)
C9—C10—C16—C18	120.36 (19)	C54—C55—C56—O3B	−10.7 (6)
C11—C10—C16—C18	−60.9 (2)	C60—C55—C56—C57	−3.1 (3)
C9—C10—C16—C17	−118.92 (18)	C54—C55—C56—C57	173.71 (17)
C11—C10—C16—C17	59.8 (2)	O3B—C56—C57—C58	−174.5 (5)
N2—C20—C21—C22	78.3 (2)	C55—C56—C57—C58	1.6 (3)
N2—C20—C21—C26	−102.40 (19)	O3B—C56—C57—C61	6.2 (5)
C26—C21—C22—O2B	−170.5 (8)	C55—C56—C57—C61	−177.76 (17)
C20—C21—C22—O2B	8.8 (9)	C56—C57—C58—C59	1.0 (3)
C26—C21—C22—C23	1.5 (3)	C61—C57—C58—C59	−179.65 (18)
C20—C21—C22—C23	−179.18 (16)	C57—C58—C59—C60	−1.9 (3)
O2B—C22—C23—C24	173.6 (7)	C57—C58—C59—C65	178.98 (19)
C21—C22—C23—C24	0.3 (3)	C56—C55—C60—O3	−179.92 (18)
O2B—C22—C23—C27	−8.7 (7)	C54—C55—C60—O3	3.3 (3)
C21—C22—C23—C27	177.93 (17)	C56—C55—C60—C59	2.1 (3)
C22—C23—C24—C25	−0.9 (3)	C54—C55—C60—C59	−174.65 (17)
C27—C23—C24—C25	−178.51 (17)	C58—C59—C60—O3	−177.80 (17)
C23—C24—C25—C26	−0.3 (3)	C65—C59—C60—O3	1.3 (3)
C23—C24—C25—C31	177.28 (17)	C58—C59—C60—C55	0.3 (3)
C22—C21—C26—O2	−179.78 (17)	C65—C59—C60—C55	179.41 (18)
C20—C21—C26—O2	0.9 (3)	C56—C57—C61—C62	−177.19 (17)
C22—C21—C26—C25	−2.7 (3)	C58—C57—C61—C62	3.5 (3)
C20—C21—C26—C25	177.94 (16)	C56—C57—C61—C64	63.5 (2)
C24—C25—C26—O2	179.19 (17)	C58—C57—C61—C64	−115.8 (2)
C31—C25—C26—O2	1.6 (3)	C56—C57—C61—C63	−56.4 (2)
C24—C25—C26—C21	2.1 (3)	C58—C57—C61—C63	124.3 (2)
C31—C25—C26—C21	−175.45 (16)	C58—C59—C65—C67	−118.5 (2)
C22—C23—C27—C28	164.4 (2)	C60—C59—C65—C67	62.4 (3)
C24—C23—C27—C28	−18.1 (3)	C58—C59—C65—C66	118.0 (2)
C22—C23—C27—C30	−77.0 (2)	C60—C59—C65—C66	−61.0 (3)
C24—C23—C27—C30	100.6 (2)	C58—C59—C65—C68	−0.8 (3)
C22—C23—C27—C29	41.6 (3)	C60—C59—C65—C68	−179.8 (2)
C24—C23—C27—C29	−140.9 (2)	C2—C1—N1—C3	72.40 (18)
C24—C25—C31—C33	122.7 (2)	C2—C1—N1—C5	−165.31 (14)
C26—C25—C31—C33	−59.8 (2)	C2—C1—N1—Pd1	−48.00 (16)
C24—C25—C31—C34	2.2 (3)	C6—C5—N1—C3	−54.56 (18)
C26—C25—C31—C34	179.64 (18)	C6—C5—N1—C1	−177.13 (14)
C24—C25—C31—C32	−116.9 (2)	C6—C5—N1—Pd1	68.57 (15)
C26—C25—C31—C32	60.6 (2)	C1—C2—N2—C4	83.83 (17)
N3—C35—C36—N4	−52.50 (19)	C1—C2—N2—C20	−158.69 (14)
N3—C39—C40—C41	−78.3 (2)	C1—C2—N2—Pd1	−30.14 (16)
N3—C39—C40—C45	98.0 (2)	C21—C20—N2—C4	175.21 (15)
C45—C40—C41—C42	−3.4 (3)	C21—C20—N2—C2	56.55 (19)
C39—C40—C41—C42	172.91 (17)	C21—C20—N2—Pd1	−67.03 (17)
C45—C40—C41—O4B	169.6 (7)	C36—C35—N3—C37	−77.92 (18)
C39—C40—C41—O4B	−14.1 (8)	C36—C35—N3—C39	163.52 (15)
C40—C41—C42—C43	0.7 (3)	C36—C35—N3—Pd2	38.17 (16)
O4B—C41—C42—C43	−173.4 (6)	C40—C39—N3—C37	−173.44 (16)
C40—C41—C42—C46B	−178.9 (7)	C40—C39—N3—C35	−53.0 (2)
O4B—C41—C42—C46B	7.0 (10)	C40—C39—N3—Pd2	67.78 (18)
C40—C41—C42—C46	−176.0 (3)	C35—C36—N4—C38	−77.38 (18)
C41—C42—C43—C44	1.4 (3)	C35—C36—N4—C54	163.50 (15)
C46B—C42—C43—C44	−178.9 (7)	C35—C36—N4—Pd2	38.72 (17)
C46—C42—C43—C44	178.0 (3)	C55—C54—N4—C38	−168.30 (15)
C42—C43—C44—C45	−0.8 (3)	C55—C54—N4—C36	−47.40 (19)
C42—C43—C44—C50	178.99 (17)	C55—C54—N4—Pd2	72.86 (16)
C41—C40—C45—O4	−178.74 (18)	C6—C11—O1—Pd1	44.6 (2)
C39—C40—C45—O4	5.0 (3)	C10—C11—O1—Pd1	−135.99 (14)

### Hydrogen-bond geometry (Å, º)

**Table:**

*D*—H···*A*	*D*—H	H···*A*	*D*···*A*	*D*—H···*A*
O5—H5···O1	0.84	1.95	2.787 (3)	171
C69—H69*A*···Cl1	0.98	2.90	3.642 (4)	134
C69—H69*C*···O3	0.98	2.57	3.267 (4)	128
O5*B*—H5*C*···O1	0.84 (2)	1.95 (3)	2.772 (6)	168 (12)
O6—H6···Cl2	0.84	2.90	3.592 (2)	141
O6—H6···Cl3	0.84	2.41	3.129 (2)	144
O6*B*—H6*B*···Cl2	0.84	2.45	3.242 (13)	157
O2—H2···O5^i^	0.84	1.88	2.715 (3)	170
O2*B*—H2*C*···Cl1	0.84	2.06	2.858 (13)	157
O3—H3···Cl1	0.84	2.46	3.1764 (17)	144
O3*B*—H3*D*···Cl3	0.84	2.12	2.886 (10)	152
O4—H4···O6^ii^	0.84	1.95	2.750 (3)	160
O4*B*—H4*D*···Cl2	0.84	2.05	2.726 (12)	137

Symmetry codes: (i) *x*, −*y*+3/2, *z*−1/2; (ii) *x*, −*y*+3/2, *z*+1/2.
